# A new transmission methodology for quality assurance in radiotherapy based on radiochromic film measurements

**DOI:** 10.1120/jacmp.v16i5.5497

**Published:** 2015-09-08

**Authors:** Leonardo L. do Amaral, Harley F. de Oliveira, Juliana F. Pavoni, Francisco Sampaio, Thomaz Ghilardi Netto

**Affiliations:** ^1^ Department of Physics Faculty of Philosophy, Sciences and Letters of Ribeirão Preto University of São Paulo Ribeirão Preto São Paulo Brazil; ^2^ Department of Internal Medicine Ribeirão Preto Medical School, University of São Paulo Ribeirão Preto São Paulo Brazil

**Keywords:** transmission quality control, radiochromic films, *in vivo* verification, *in vivo* dosimetry, quality assurance

## Abstract

Despite individual quality assurance (QA) being recommended for complex techniques in radiotherapy (RT) treatment, the possibility of errors in dose delivery during therapeutic application has been verified. Therefore, it is fundamentally important to conduct *in vivo* QA during treatment. This work presents an *in vivo* transmission quality control methodology, using radiochromic film (RCF) coupled to the linear accelerator (linac) accessory holder. This QA methodology compares the dose distribution measured by the film in the linac accessory holder with the dose distribution expected by the treatment planning software. The calculated dose distribution is obtained in the coronal and central plane of a phantom with the same dimensions of the acrylic support used for positioning the film but in a source‐to‐detector distance (SDD) of 100 cm, as a result of transferring the IMRT plan in question with all the fields positioned with the gantry vertically, that is, perpendicular to the phantom. To validate this procedure, first of all a Monte Carlo simulation using PENELOPE code was done to evaluate the differences between the dose distributions measured by the film in a SDD of 56.8 cm and 100 cm. After that, several simple dose distribution tests were evaluated using the proposed methodology, and finally a study using IMRT treatments was done. In the Monte Carlo simulation, the mean percentage of points approved in the gamma function comparing the dose distribution acquired in the two SDDs were 99.92%±0.14%. In the simple dose distribution tests, the mean percentage of points approved in the gamma function were 99.85%±0.26% and the mean percentage differences in the normalization point doses were −1.41%. The transmission methodology was approved in 24 of 25 IMRT test irradiations. Based on these results, it can be concluded that the proposed methodology using RCFs can be applied for *in vivo* QA in RT treatments.

PACS number: 87.55.Qr, 87.55.km, 87.55.N‐

## I. INTRODUCTION

The main tests in the quality assurance (QA) program of radiotherapy (RT) equipment check its mechanical and dosimetric parameters to ensure an adequate treatment delivery. However, in high technology treatments it is also necessary to evaluate the dose distribution of each individual planning, in addition to the absolute doses delivered.^(1)^ For such evaluation, it is necessary the use of suitable detectors to achieve the measurement objectives. Some of the most commonly used detectors are the two‐dimensional (2D) arrays of detectors and dosimetric films, in association with the ionization chambers.

Two‐dimensional arrays of detectors are arrays of detectors capable of acquiring a dose map or a dose distribution in two dimensions in only one exposure. These devices are widely used because of its simple handling; however, there are difficulties related to their spatial and angular dependence.^2^, ^3^

Dosimetric films are widely used in clinical dosimetry; its main advantage is the high spatial resolution^(4)^ in 2D dose distribution measurements. Two types of dosimetric films can be mentioned: radiographic films and radiochromic films (RCFs). Radiographic dosimetric films have been used for a long time, but their results can be influenced by temperature variations or contamination in the development process. Currently, RCFs that do not present this limitation are replacing radiographic dosimetric films. RCFs change their coloration by the absorption of energetic radiation without requiring latent chemical, optical or thermal development, or amplification. The main advantages of the current RCFs are: density equivalence to water, high spatial resolution,^(5)^ high dose response range, minimum energy dependence and, especially, its insensitivity to visible light.^(6)^

Despite individual QA being recommended for complex techniques in RT treatment, the possibility of errors in dose delivery during therapeutic application has been verified.^(7)^ With the increasing complexity of treatments, especially with the advent of intensity‐modulated radiation therapy (IMRT), many studies have reiterated the need for an appropriate QA program. Mans and colleagues^(8)^ presented the results of an *in vivo* evaluation of 4337 treatments of IMRT and concluded that the individual QA of the patient plan before treatment is not sufficient to ensure the quality of treatment, considering that 17 errors during treatment application were found in this series. Therefore, it is fundamentally important to conduct *in vivo* QA during treatment.^(7)^

The *in vivo* evaluation is intended to ascertain if the dose is released at the correct location and with the correct intensity. In teletherapy, this is generally performed by placing a dosimeter on the patient's skin at the beam entrance; for more complex techniques, however, such methodology is subject to some difficulties.^7^, ^9^ In radiosurgery and stereotactic radiotherapy, for example, techniques that use various noncoplanar fields with reduced dimensions or rotational beams, there is a difficulty in finding the correct point on the patient's skin to position the dosimeters for beam entrance doses measurement. Furthermore, the time required for this procedure may be a limitation for achieving this control. For IMRT, in particular, the biggest concerns are the methods of dose distribution analysis for each field at the position of the dosimeters placed on the patient's skin, as the surface irregularity added to beam intensity modulation makes such analysis difficult.^(7)^

In view of the need for dose assessment at the time of therapy and that the *in vivo* evaluation is not well established for more complex treatment techniques, several researchers have dedicated themselves to developing QA and *in vivo* dosimetry methods using an electronic portal imaging device (EPID).^8^, ^10^ These devices are coupled only to some linear accelerators (linacs) and it is possible to use linacs without EPIDs for these special treatment techniques. In addition, even for linacs equipped with EPID, its use for *in vivo* dosimetry is not guaranteed, since this procedure is not yet well established for all devices manufacturers and, also, a specific calibration for dosimetry purposes is necessary.^(11)^

The first independent methodology to review the monitor units (MU) of a treatment plan in a noncomputerized linac was described by Paliwal and colleagues.^(12)^ Poppe et al.^(13)^ described and evaluated a technique using an ionization chamber matrix coupled to the linac treatment head in order to conduct a pretreatment QA, as well as for therapeutic applications. McCurdy and colleagues^(14)^ tested MatriXX (IBA Dosimetry, Schwarzenbruck, Germany), a bidimensional array of ionization chambers coupled to the linac treatment head for performing IMRT QA. All of these studies are examples of transmission QA methodologies; they present the main advantage of coupling the measurement device to the linac treatment head, making the beam always perpendicular to the device's sensitive volume, eliminating variations in its response as a function of the gantry's angular displacement. Another important advantage is that, depending on the detector, this methodology can be used for *in vivo* evaluation.^15^, ^16^, ^17^, ^18^

One of the most recent publications in this field presents a multiwire or multistrip transmission ionization chamber. In this methodology, the wires are placed between two polycarbonate windows with a total thickness of 1.4 cm and subjected to a potential difference of 400 V. During irradiation, different lengths and pairs of wires are exposed and the produced ions are collected by an electrometer.^(13)^ With a similar system, Page et al.^(16)^ also conducted tests to evaluate *in vivo* dosimetry, demonstrating that it can be achieved by placing an ionization chamber system using a thin monolithic active pixel sensor (MAPS). With the same goal, Amaral and colleagues^(19)^ conducted a study inserting RCF segments at the head of a linac to assess exposures in stereotactic radiotherapy. They were able to formulate a methodology for *in vivo* dose verification by assessing RCF segments relative densities.

Although several studies are developing devices for dosimetric transmission QA, no study has been published so far describing a methodology using RCFs, and also, it is not well established any *in vivo* simple dosimetric methodology for evaluation of complex treatment techniques in RT. Thus, the aim of this work is to present a simple methodology of transmission QA using RCF coupled to the linac accessory holder in its treatment head for *in vivo* verification of complex treatment techniques in RT.

## II. MATERIALS AND METHODS

### A. General parameters of the proposed methodology

**Figure 1:**
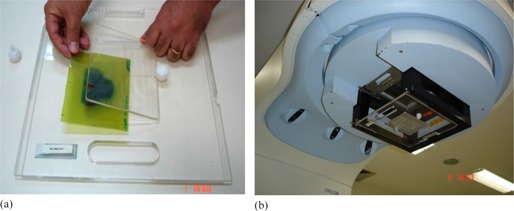
The figures show (a) the acrylic support used, and (b) the acrylic support coupled to the linac accessory holder.

The QA methods developed in this work were based on obtaining the *in vivo* and 2D radiation dose distribution of complex RT treatment plans using Gafchromic EBT2 films (ISP Corporation, Wayne, NJ). The film was positioned on an acrylic support (similar to a tray) with a source‐to‐detector distance (SDD) of 56.8 cm, attached to the linac accessory holder (ONCOR Impression; Siemens, Erlangen, Germany) throughout the delivery of the planning irradiation fields (Figs. 1(a) and (b)).

**Figure 2:**
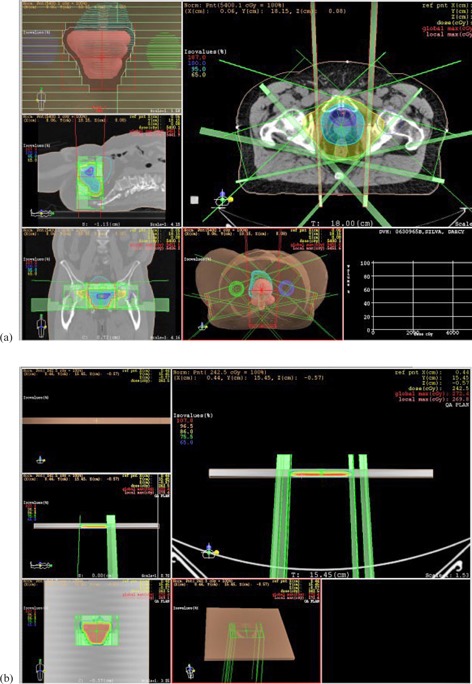
(a) Image of a treatment planning prostate case in the TPS. (b) Setting of the dose distribution calculated by TPS on the phantom with dimensions similar to the acrylic support, in yellow the DD calculated on the coronal plane can be observed.

Twenty‐four hours after irradiation, the film was scanned (Vidar DiagnosticPRO Advantage, Herndon, VA) and a calibration for the expected absorbed dose using the treatment planning system (TPS) was applied to the pixel number values. Finally, a gamma analysis (3% dose difference, 3 mm distance‐to‐agreement criteria, 10% threshold) was conducted to compare the radiation dose distribution being measured to that one calculated with the XiO TPS (v. 3.62, Elekta, Stockholm, Sweden). The calculated dose distribution is obtained in the coronal and central plane of a phantom with dimensions of 30cm×30cm×1cm positioned with a 100 cm SDD as a result of transferring the actual IMRT plan with all the fields positioned with the gantry vertically, that is, perpendicular to the phantom (Figs. 2(a) and (b)).

The transmission QA was considered approved when it was obtained simultaneously a dose difference in the normalization point of less than 5% and a percentage of points approved in the gamma test exceeding 95%.

#### A.1 Acrylic support description and calibration methodology

The acrylic support used has dimensions of 30cm×30cm×1cm and has a socket in its center with dimensions of 20cm×15cm×0.5cm for film placement. It is covered by an acrylic plate with similar dimensions and both are secured by two screws to prevent them from falling when the gantry is tilted (Fig. 1(a)). The support transmission for the 6 MV beam was 94.2%.

To allow the comparison of the dose distribution measured with film to the dose distribution expected by the TPS a calibration curve is necessary. This calibration methodology is one of the main points of the proposed methodology, since it allows the conversion of the film pixel values measured when irradiating the film at the accessory holder (SDD=56.8 cm) to the TPS calculated absorbed doses (SDD=100 cm).

**Figure 3:**
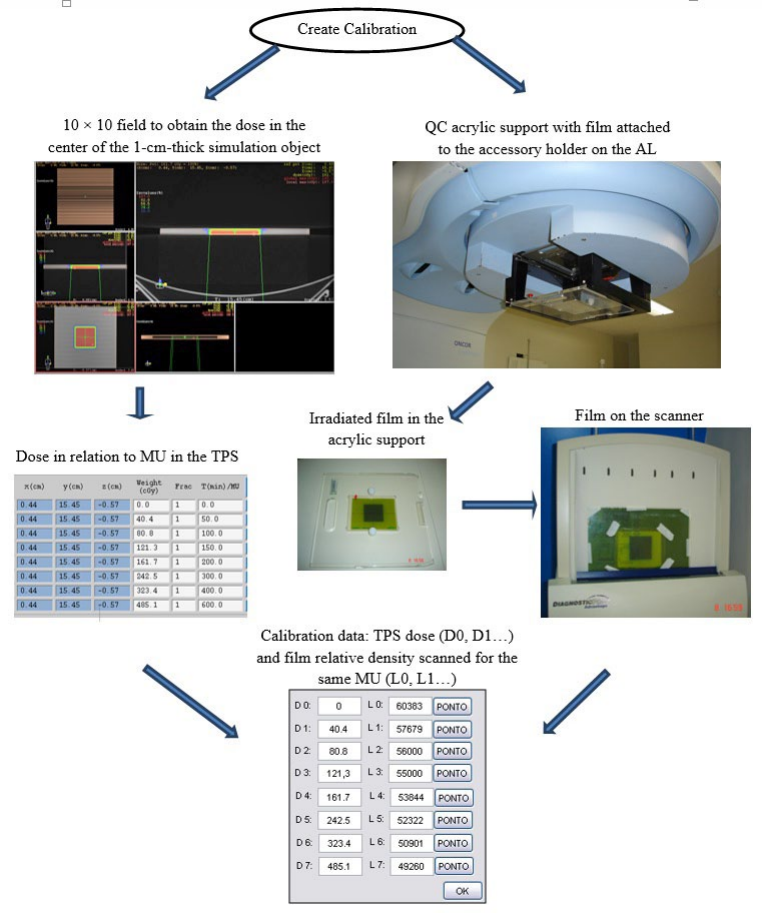
Calibration scheme describing the methodology of QA with film on acrylic support: (left) setting used to acquire the dose calculated by TPS; (right) setting used to acquire scanned image pixel values of the films irradiated in the acrylic support. In the bottom of the image, the calibration relation is created associating the TPS calculated dose with the corresponding pixel value obtained in the irradiated film.

The calibration was performed by irradiating the RCF on the support attached to the linac accessory holder (SDD=56.8 cm) with 50, 100, 200, 400, and 600 monitor units (MUs) and using a field size of 10 cm×10 cm. The films were scanned 24 hrs after irradiation, at a resolution of 75 dpi and 12 bits. The obtained pixel value results were associated with the respective absorbed doses calculated by the TPS in the center of the phantom, with dimensions similar to the acrylic support at a SDD of 100 cm and by delivering the same MU used for film irradiation. The calibration was applied grouping the number pixel values of the scanned film, and the dose values obtained through the TPS with the same MU that were irradiated onto the film (Fig. 3).

Another important point to be highlighted here is the film setup during scanning for calibration or for dose distribution analysis; this procedure is essential to avoid influences in the result, such as polarization effects. After an extensive study of different film setups during scanning,^(20)^ the best setup found and the one used in this study used the RCF fixed in a support film with a transparent film covering it and selecting a region of interest (ROI) for scanning.

### B. Transmission methodology validation

To validate the proposed transmission methodology, a Monte Carlo simulation was used to verify the difference between the dose distribution generated at a SDD of 56.8 cm (the source distance where the film was positioned during measurement) and 100 cm (the source distance where the TPS calculated the expected dose distribution). Then, several dose distribution comparisons for controlled configurations using the measured dose distribution obtained with the RCF in the accessory holder (SDD=56.8 cm) and the calculated dose distribution obtained with the TPS (SDD=100 cm) were done to prove the methodology. The first set of tested configurations was composed of square fields, the second was an irregular field, and the third was a five‐fields merging of a conformal prostate cancer treatment. Finally, the methodology was evaluated using a more complex treatment technique, an IMRT treatment irradiation.

#### B.1 Monte Carlo simulation

To evaluate the feasibility of the transmission QA technique proposed, a Monte Carlo simulation was done using PENELOPE code (Penetration and Energy Loss of Electrons and Positron), version 200819.^(21)^ The application of this tool aimed to investigate the differences between the dose distribution obtained for the two SDDs used for the same field size (100 cm and 56.8 cm). These dose distributions should be the same for the success in the application of the proposed methodology; if they are different, the proposed comparison of the film measurement (SSD=56.8 cm) to the expected dose distribution calculated by the TPS (SDD=100 cm) would be different due to the beam differences and not due to any mistake during the application of the treatment, that is the main objective of the proposed methodology. In order to reproduce the experiment, the ONCOR linac treatment head was simulated and the photon beam spectra of the 6 MV beam for the different field sizes was achieved and analyzed.^22^, ^23^

To verify if the generated spectrum agreed with the linac used in this work, three simulations were done using field sizes of 5 cm×5 cm,10 cm×10 cm, and 15 cm×15 cm and $1.10^{9}$ stories. These simulations were performed in a water phantom with dimensions of 40cm×40cm×40cm at a distance of 100 cm from the source, and the simulated percentage depth doses (PDD) were compared with PDD measured with a CC13 ionization chamber (IBA Dosimetry) in the 6 MV beam of the ONCOR linac, in the same configuration.

Then, new simulations with $1.10^{9}$ stories were performed to evaluate the dose deposition in the two SDDs mentioned (100 cm and 56.8 cm) and to generate their respective dose distribution in the coronal plane of the acrylic phantom with 1 cm thick used in the methodology. A gamma function was carried out to compare the dose distribution generated at SDD of 56.8 cm in the acrylic support that holds the film with the dose distribution generated at SDD=100 cm, the position of the acrylic phantom used in the TPS. It used the maximum dose percentage variation of 3%, maximum distance variation of 3 mm, and threshold of 10%. To implement the gamma function, the dose distribution obtained with SDD=56.8 cm was magnified to the size of the dose distribution obtained with SDD=100 cm and, after that, a manual coregistration based on the agreement of both dose distributions in two perpendicular planes was done. This process was performed in an in‐house software developed using MATLAB (MathWorks, Natick, MA). Furthermore, a Gaussian convolution was also applied to the dose distribution to reduce noise in the data. Also, a linear interpolation was done to quadruple the dose distribution points, improving the dose distribution coregistration and increasing the amount of data for the evaluation. This quadruplication reduced the distance between the points in the dose distribution from 2 mm to 0.5 mm.

#### B.2 Controlled configurations for DD comparisons

In order to evaluate the difference between irradiated films in the proposed configuration and the dose distribution generated by TPS (SDD=100 cm), several controlled irradiation configurations were tested. The first set of configurations was composed of square fields; three films were irradiated with 5 cm×5 cm,10 cm×10 cm and 15 cm×15 cm square fields. The films were irradiated using 200 MU and the scanned dose distributions were compared to the TPS‐calculated dose distribution. These films were magnified using ImageJ software (WS Rasband, Bethesda) to achieve the same resolution of the calculated dose distribution, allowing a comparison using the gamma function. The gamma function was performed in the OmniPro I'm*RT* software (IBA Dosimetry) with the following criteria: 3% of maximum dose variation, 3% of distance maximum variation, and threshold of 20%.

The second tested configuration was an irregular field that was one of the fields of a conformal prostate treatment. This irregular field was first transferred to the CT image of the phantom used in the TPS and the calculated dose distribution was obtained. Then, the film was irradiated at the acrylic support in the linac accessory holder and 24 hrs after irradiation, it was scanned. The gamma function comparing both dose distributions was again performed using OmniPro I'm*RT* software with the same criteria described above.

The third tested configuration was a complete five coplanar field conformal prostate cancer treatment with gantry angles of 180°, 260°, 315°, 45°, and 100°. All of this prostate treatment simulation was transferred to the CT image of the phantom used in the TPS; however, at this transfer, all gantry angles were modified to 0°, being all beams perpendicular to the phantom in the same manner as to the film. This modification is necessary because, at the time of irradiation, the irradiated film in the acrylic support is always perpendicular to the beam, being necessary to use the same geometry for the TPS dose distribution generation in order to be comparable to the film measured dose distribution. For this evaluation, the same film was irradiated with all treatment fields. The gamma function was applied comparing the scanned dose distribution measured with the film and the dose distribution generated by the TPS, using the same criteria as described above.

The fourth set of configurations was composed of five IMRT plans to validate the methodology in a more complex treatment technique. Each plan was assessed using five different configurations. For the first two, all the irradiations were conducted with the gantry at 0°; they used the same five IMRT plans, but the QAs identified as set 2 were performed one week after the QAs of set 1. For the third plan, the irradiations were conducted with actual planning gantry angles. For the last two plans, the irradiations were conducted using actual gantry angles; however, for the fourth one, fewer monitor units than at the real plan were used, and for the fifth one, one of the fields was removed. This way, the first three irradiations were expected to pass in the QA and the last two irradiations were expected to fail, since they were simulated with known errors. An aspect to be highlighted is that, despite the several IMRT gantry angles being used for the treatment plans, the dose distributions used for comparison with the irradiated film must be obtained in the TPS with all gantry angles placed at 0° because the film attached to the acrylic support will always remain perpendicular to the beam.

### C. Other studies to validate the transmission methodology

#### C.1 Scanning reproducibility

In order to verify the reproducibility using the film scanning technique, a series with 10 QAs was performed through the proposed methodology and the results were analyzed using two scans for the same film at different times. The reproducibility assessment was conducted by comparing the dose difference at the normalization point and the gamma function of the two scans.

#### C.2 QA reproducibility with the film on the acrylic support

In order to verify the reproducibility of the proposed QA technique, this methodology was repeated twice in the QA of five selected IMRT plans. The reproducibility assessment was conducted by comparing the dose difference at the normalization point and the gamma function of the two irradiations of the same planning.

#### C.3 Comparison between the QA with film on the acrylic support and QA using other dosimetry systems

The proposed QA was compared to the QA formed by two conventional dosimetry systems: the MatriXX with solid water plates to provide sufficient backscattering, and the RCF positioned over the MatriXX and below the solid water plates. These QA comparisons were performed using the five IMRT plans of the fourth set of controlled configurations. With each irradiation, the three dosimeters were irradiated together, all at 0° field angle. This was done to avoid influences of the linac performance in our comparisons. For the conventional QA, the gamma analysis were obtained through OmniPRO software (OmniPro‐I'm*RT*), taking into consideration the attenuation by the acrylic support. For the film positioned over MatriXX, a calibration based on known absorbed dose values was also conducted.

## III. RESULTS

### A. Transmission methodology validation

#### A.1 Monte Carlo simulation

The mean percentage deviations between the measured and simulated PDP for the 5 cm×5 cm field size was 0.52%±0.43%; for the 10 cm×10 cm field size it was 0.78%±0.69% with only one deviation greater than 1.5%; and finally, for the 15 cm×15 cm field size it was 0.67%±0.75% with only one percent deviation exceeding 2%. For our purpose, these results validate the simulated spectrum for the studied fields.

The percentage of points approved in the gamma function comparing the dose distribution in the SDD of 100 cm and 56.8 cm was 100% for the 5 cm×5 cm field size, 99.9% for the 10 cm×10 cm field size, and 99.75% for the 15 cm×15 cm field size.

#### A.2 Controlled configurations for DD comparisons between TPS calculation and film measurement

Table 1 presents the first three sets of controlled configurations tested using the proposed transmission QA methodology.

**Table 1:** Results comparison of TPS‐calculated dose distribution and film‐measured dose distribution for the first three sets of controlled configurations tested

*Irradiation Configuration*	*Gamma (%)* ^a^	*Film (cGy)* ^b^	*TPS (cGy)* ^c^	*Point Dose Deviation*
Square field: 5 cm×5 cm	100.0	150.3	156.7	−4.1
Square field: 10 cm×10 cm	99.9	168.0	168.9	−0.5
Square field: 15 cm×15 cm	99.4	178.2	175.3	1.6
Irregular Field	99.9	155.9	163.0	−4.3
5 merged conformal fields	99.9	250.3	249.6	0.3

a Percentage of points approved in the gamma function

b Dose in the normalization point of film dose distribution

c Dose in the normalization point of TPS dose distribution

d Punctual deviation between dose in the normalization point of TPS dose distribution and film dose distribution

Table 2 presents the data for the fourth set of controlled configurations tested. In this test, the QA methodology was conducted in five IMRT treatment with the variations described above. It can be observed that the proposed QA was in agreement with 24 of the 25 configurations tested — that is, 96% of the results.

**Table 2:** Proposed QA results for the fourth set of controlled configuration grouped in accordance with the irradiation

	*Planninga*	*XiO Dose* ^b^	*Film Dose* ^c^	*%Diff* ^d^	*Gamma* ^e^	*Result* ^f^
*Quality Control to Pass*						
1^st^ 0° Gantry Irradiation	1	245.6	247.5	0.8	99.9	Pass
	2	185.7	176.9	−4.7	99.0	Pass
	3	253.0	253.0	0.0	98.4	Pass
	4	230.2	230.0	−0.1	98.9	Pass
	5	207.5	198.7	−4.2	96.8	Pass
2^nd^ 0° Gantry Irradiation	1	245.3	242.2	−1.3	98.3	Pass
	2	185.7	182.0	−2.0	99.7	Pass
	3	255.9	248.5	−2.9	97.7	Pass
	4	230.2	232.8	1.1	100	Pass
	5	197.9	191.5	−3.2	96.6	Pass
3^rd^ Real Gantry Angle Irradiation	1	245.9	250.7	2.0	97.9	Pass
	2	173.5	166.7	−3.9	96.7	Pass
	3	252.8	252.9	0.0	98.6	Pass
	4	230.2	230.1	0.0	99.4	Pass
	5	207.5	195.9	−5.6	95.5	Fail
*Quality Control to Fail*						
1^st^ Lower MU Irradiation	1	245.8	217.8	−11.4	96.9	Fail
	2	173.5	137.5	−20.7	99.7	Fail
	3	252.6	159.6	−36.8	98.2	Fail
	4	230.2	197.1	−14.4	97.1	Fail
	5	208.7	165.8	−20.6	99.8	Fail
2^nd^ 1 field Removed Irradiation	1	245.7	220.1	−10.4	92.9	Fail
	2	185.7	148.9	−19.8	99.5	Fail
	3	252.8	226.4	−10.4	47.3	Fail
	4	230.2	219.6	−4.6	51.0	Fail
	5	208.5	159.1	−23.7	70.2	Fail

a Planning that is being analyzed.

b Dose in the XiO planning at the normalization point.

c Dose obtained in the film at the normalization point.

d Percentage difference of the doses measured by the film and calculated by the TPS at the normalization point.

e Percentage of points passing in the gamma function.

f QA result of Pass or Fail according to the criteria adopted.

### B. Other studies to validate the transmission methodology

#### B.1 Scanning reproducibility

The scanning reproducibility results presented a mean dose difference at the normalization point of 0.20%±0.66%, and the mean difference found between the gamma functions for the different scans was −0.37%±0.90%.

#### B.2 QA Reproducibility with the film on the acrylic support

The mean difference of the dose at the normalization point between the two irradiations was −0.01%±2.37%, and the mean difference for the gamma functions between irradiations was 0.14%±1.08%.

#### B.3 Comparison between the QA with film on the acrylic support and QA using other dosimetry systems

In the comparison between the QA with film on the acrylic support and the MatriXX, the gamma function mean difference was −0.22%±2.95%. Furthermore, the percentage difference between the gamma functions between the acrylic support film and the film above the MatriXX was −1.06%±0.67%.

## IV. DISCUSSION

Based on the Monte Carlo simulation and on the results found in comparisons between the film dose distribution and the TPS calculated dose distribution, it is suggested that the beam does not change significantly for the geometry employed in this work when the SDD varies from 56.8 cm to 100 cm. Thus the proposed method for transmission QA with the dosimeter attached to the linac accessory holder can be employed.

In the first three sets of controlled configuration tested, all percentage of points approved in the gamma function are of the same order of the ones found using Monte Carlo simulation, showing again that the dose distribution measured with film in the linac accessory holder and the TPS calculated dose distribution are comparable.

In the fourth set of controlled configuration tested, there were 15 correct IMRT treatment simulations and 10 configurations with some deviation in the administered dose, in order to verify whether the proposed methodology would be capable of identifying such deviations. The proposed QA was in agreement with the expectations in 24 simulations, and only one of the results was not. This represents a concordance of 96% with expectations, validating the proposed methodology. The QA failure in one of the configurations (planning number 5 of the third set of irradiations) does not indicate that the proposed methodology is flawed. The difference found can be attributed to a small fluctuation in the linac response at the time of irradiation and just emphasizes the need of an *in vivo* verification of the treatment delivered to detect such deviations.

The purpose of the QA methodology proposed in this work is to verify *in vivo* the dose distribution delivered by the linac, subsequent to conventional QA — that is, it is assumed that the treatment plan being assessed has already passed the individual planning QA and thus it must pass the *in vivo* QA. This way, in case the test does not pass with the proposed methodology, the user cannot cancel out the plan immediately, but on the contrary, it must be reassessed.

The methodology used to scan the film proved to be reproducible for two scans on the same film and also the QA reproducibility was achieved in the analyses with two exposures for the same planning. For the two tests, the fluctuations revealed are considered statistical fluctuations.

The comparisons of the results obtained with the proposed methodology and with the QA conducted using other dosimeters already established for this application were also satisfactory, which validates the proposed methodology. Only the standard deviation of 2.95% found between the QA performed with the MatriXX and that suggested by our work is noteworthy; however, such difference can be explained by the MatriXX low spatial resolution and large detector dimensions.^(2,3)^

This QA methodology using RCF on the accessory holder at the radiation beam output must be applied by observing that the acrylic support used. In this study, it absorbs 5.8% of the dose, which requires a correction in the exposition to be applied. However, this factor does not limit the use of this technique because the dose being applied can be corrected. Furthermore, such attenuation is similar to that provided by PTW's DAVID,^(13)^ and it is below that of the method proposed using the MatriXX.^(24)^ In the proposed methodology, it is also possible to refine the procedure by designing a support with lower attenuation.

During all the irradiations done in this work, an acrylic support with holes in the place of the contact with the interlock sensors was used; this way the linac didn't recognize its presence.

However, for safety reasons, when using it for patient verification, the staff must consider using the support as a tray and account for its absorption factor.

One limitation of this QA technique in IMRT is that it does not assess the patient's position. Therefore, this methodology must be associated with other control modalities, such as portal film or IGRT techniques, to determine the position.

This work is important for enabling an *in vivo* verification methodology that uses low‐cost commercial dosimeters usually available in radiation therapy departments. Considering that this methodology does not present any dependence with the gantry angle, it can be applied to the most modern RT techniques, such as volumetric‐modulated arc therapy (V‐MAT) using rotational beams. The methodology using the EBT2 RCF attached to the gantry linac accessory holder is effective for *in vivo* QA in IMRT.

## V. CONCLUSIONS

In this study, we proposed the creation of a simple and low‐cost methodology for transmission dosimetry using RCF dosimetry. By the exhaustive set of controlled irradiations tests, it can be concluded that the *in vivo* QA methodology using a RCF attached to the linac accessory holder in RT applications is efficient for *in vivo* verification of IMRT treatments. Moreover, this methodology does not present any dependence with the gantry angle, and can be applied to the most modern RT techniques, such as volumetric‐modulated arc therapy (V‐MAT) using rotational beams.
